# Metagenome-Assembled Genomes from Murine Fecal Microbiomes Dominated by Uncharacterized Bacteria

**DOI:** 10.1128/mra.01162-22

**Published:** 2023-02-13

**Authors:** Kiarash Rastegar, Scott T. Kelley, Varykina G. Thackray

**Affiliations:** a Bioinformatics and Medical Informatics Program, San Diego State University, San Diego, California, USA; b Department of Biology, San Diego State University, San Diego, California, USA; c Department of Obstetrics, Gynecology, and Reproductive Sciences, University of California, San Diego, La Jolla, California, USA; Wellesley College

## Abstract

The laboratory mouse gut microbiome has been extensively studied, but our understanding of its diversity remains incomplete. We report the assembly of 51 draft metagenome-assembled genomes (MAGs) from murine fecal samples dominated by uncharacterized bacteria. These MAGs add to our understanding of gut microbial diversity in this critical model organism.

## ANNOUNCEMENT

Murine models are an important tool for studying the role of the microbiome in different aspects of mammalian physiology and disease (1–3). Our research has explored the relationship of the gut microbiome to sex steroid hormones, with a particular focus on the hyperandrogenism that occurs in polycystic ovary syndrome (4–6). Here, we report on metagenome-assembled genome (MAG) assemblies from two murine fecal metagenomes dominated by uncharacterized bacteria. Three-week-old C57BL/6N female mice were purchased from Envigo and housed in a vivarium at the University of California, San Diego, with an automatic 12-h light/12-h darkness cycle (light period from 6:00 a.m. to 6:00 p.m.). Mice were given *ad libitum* access to water and food (Teklad global 18% protein extruded diet; Envigo). Fecal samples were collected in the morning at 7 weeks of age. DNA was extracted from ~100-mg frozen murine fecal samples using the DNeasy PowerSoil kit (Qiagen) according to the manufacturer’s instructions, and extracted DNA was stored at −80°C. Libraries were prepared and sequenced on an Illumina NovaSeq system at the University of California, San Diego Institute for Genomic Medicine Genomics Center. Specifically, 100 ng of genomic DNA was sonicated using an E220 focused ultrasonicator (Covaris) to produce 600-bp fragments, which were purified using Agencourt AMPure XP beads (Beckman Coulter). A KAPA HyperPrep kit (Kapa Biosystems) was used to prepare Illumina libraries following the manufacturer's instructions. Libraries were quality checked for their size and concentration with electrophoresis using a high-sensitivity D1000 kit on a 2200 TapeStation (Agilent). Sequencing generated a total of 130.58 million reads combined for the two samples (60.64 million reads and 69.94 million reads), and the resulting paired-end metagenomic fastq files were trimmed and quality controlled using fastp v0.12.4 (7). The fastp program trimmed adapters, filtered out low-quality reads, excised poor-quality bases from the 5′ and 3′ ends, and produced forward and reverse sequences 100 nucleotides in length for further analysis. Assembly of the metagenomes was performed with metaSPAdes v3.15.3 (8). The contigs were uploaded to KBase (9) and further analyzed with the MaxBin 2.0 v2.2.4 (10) binning algorithm. These bins were then further refined using DAS Tool v1.1.2 (11), which collapsed the number of bins from 116 to 51. The DAS Tool bins were then annotated using the microbial genome annotator tool RASTtk v1.073 (12). Once the annotations were complete, we used GTDB-Tk v1.7.0 (13) to taxonomically classify the bins. To check the composition of the genome, we used CheckM v1.0.18 (14), which shows the quality of the bins and how many single-copy genes they contain. To determine which bins were the most abundant in the metagenomes, we used Salmon v1.8.0 (15) to measure the abundance of the contigs in each bin by mapping the reads to the assembled contigs and determining how many times the contigs aligned to the reads. The two read libraries were combined before the Salmon mapping process. All tools used in the analysis were run with default parameters unless otherwise specified.

**FIG 1:**
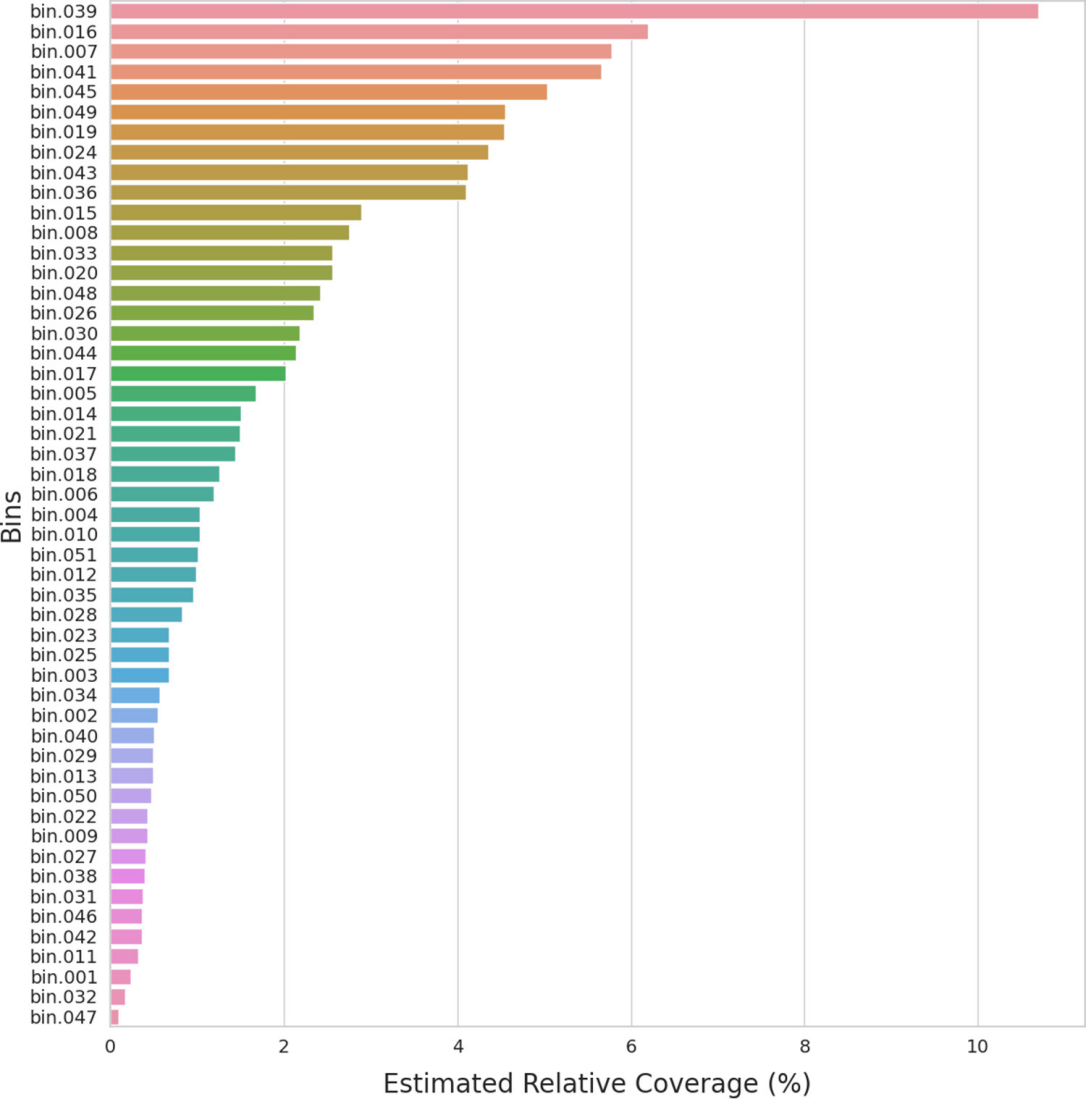
Relative abundance in the most abundant bins. The relative abundance is the number of reads per bin divided by the total number of reads in all of the KBase bins from both metagenome samples. The bin numbers can be found next to the NCBI identifications in Table 1. The most abundant bin is bin 039, a *Muribaculum* sp. strain.

Table 1 details information on the bins, including the GenBank accession numbers, the closest genome in the Genome Taxonomy Database (GTDB), the completeness of the genomes, the number of contigs, and the NCBI-assigned taxonomy. The KBase workflow also produced a plot of MAG genome quality, a phylogenetic analysis of the MAGs, and a visualization of their functional pathways (16, 17). The Salmon analysis determined the relative abundances of the MAGs (Fig. 1). The most abundant MAG according to the Salmon analysis was a novel *Muribaculum* sp. strain (formerly S24-7) (bin 039) (Fig. 1 and Table 1).

**TABLE 1:** Accession numbers, assembly statistics, and taxonomic assignments for MAGs

GenBank accession no.^*a*^	GenBank assembly accession no. for FastANI reference^*b*^	FastANI ANI (%)^*c*^	Genome size (bp)	GC content (%)	Avg coverage (%)	Completeness (%)^*d*^	Contamination (%)^*e*^	No. of contigs^*f*^	*N*_50_ (bp)^*g*^	NCBI organism name and bin number^*h*^
JAPKVN010000000			1,103,159	49.41	0.24	60.34	2.93	96	67,037	*Clostridia* bacterium, bin 001
JAPKVO010000000	GCF_000012845.1	97.32	4,373,110	45.31	0.54	94.10	1.67	131	62,302	Parabacteroides distasonis, bin 002
JAPKVP000000000	GCF_003762875.1	96.16	3,022,371	54.04	0.67	95.74	27.8	122	51,464	*Duncaniella* sp., bin 003
JAPKVQ000000000			4,108,057	55.39	1.03	75.35	10.3	219	43,157	*Dysosmobacter* sp., bin 004
JAPKVR000000000			1,786,207	43.74	1.68	95.16	<0.01	91	41,188	*Clostridia* bacterium, bin 005
JAPKVS000000000			2,212,258	30.52	1.20	91.57	8.43	158	21,184	*Bacillus* bacterium, bin 006
JAPKVT010000000	GCF_000487995.1	99.94	2,331,977	31.48	5.77	98.28	2.59	21	127,566	Mucispirillum schaedleri, bin 007
JAPKVU010000000	GCA_009774395.1	98.61	5,946,436	39.21	2.75	95.15	8.35	81	103,071	*Lachnospiraceae* bacterium, bin 008
JAPKVV010000000			2,068,202	51.65	0.42	99.04	0.51	126	94,090	*Rikenellaceae* bacterium, bin 009
JAPKVW010000000			3,247,007	48.90	1.03	97.65	1.34	33	85,270	*Oscillospiraceae* bacterium, bin 010
JAPKVX010000000			2,298,632	41.29	0.32	88.97	12.3	56	77,641	*Clostridia* bacterium, bin 011
JAPKVY010000000			1,825,488	43.67	0.99	95.70	2.15	62	197,687	*Alphaproteobacteria* bacterium, bin 012
JAPKVZ010000000			2,898,828	47.09	0.49	77.29	1.68	102	45,504	*Lachnospiraceae* bacterium, bin 013
JAPKWA010000000	GCF_000403275.1	98.82	3,584,479	45.43	1.51	96.26	4.70	103	59,311	*Kineothrix* sp., bin 014
JAPKWB010000000	GCF_000403495.2	98.42	4,637,001	40.24	2.89	97.13	3.55	142	25,930	*Lachnospiraceae* bacterium, bin 015
JAPKWC010000000			3,070,752	48.32	6.20	96.43	0.48	74	101,673	*Bacteroidales* bacterium, bin 016
JAPKWD010000000	GCA_009774765.1	99.96	2,706,227	46.62	2.02	98.17	1.19	194	43,433	*Bacteroidales* bacterium, bin 017
JAPKWE010000000	GCA_001689535.1	98.61	1,836,513	54.39	1.25	65.28	3.84	432	48,531	*Paramuribaculum* sp., bin 018
JAPKWF010000000			4,495,251	48.39	4.54	97.27	3.62	96	52,116	*Lachnospiraceae* bacterium, bin 019
JAPKWG010000000	GCA_002490725.1	99.97	1,581,772	56.66	2.56	74.53	0.75	98	48,943	*Muribaculaceae* bacterium, bin 020
JAPKWH010000000	GCF_003024925.1	99.39	1,954,743	55.72	1.50	87.17	0.38	580	27,976	Paramuribaculum intestinale, bin 021
JAPKWI010000000	GCF_003612565.1	99.23	3,068,355	44.52	0.42	88.96	3.09	29	90,657	*Lachnospiraceae* bacterium, bin 022
JAPKWJ010000000	GCA_009775535.1	99.25	1,524,160	50.48	0.68	69.25	0.38	108	44,576	*Muribaculaceae* bacterium, bin 023
JAPKWK010000000			3,481,771	37.50	4.36	98.31	1.54	135	50,027	*Lachnospiraceae* bacterium, bin 024
JAPKWL010000000			1,861,201	27.30	0.67	86.02	1.29	272	59,996	*Clostridia* bacterium, bin 025
JAPKWM010000000			4,400,590	51.07	2.34	90.73	0.92	41	126,016	*Lachnospiraceae* bacterium, bin 026
JAPKWN010000000	GCA_009773975.1	99.81	2,383,586	52.87	0.41	87.49	0.59	113	83,350	*Desulfovibrio* sp., bin 027
JAPKWO010000000	GCA_003979135.1	98.75	2,060,160	54.96	0.83	99.90	2.04	64	99,201	*Alistipes* sp., bin 028
JAPKWP010000000			2,714,132	52.97	0.50	97.18	25.8	59	61,622	*Clostridia* bacterium, bin 029
JAPKWQ010000000			5,217,971	51.80	2.18	98.25	34.2	610	24,850	*Acetatifactor* sp., bin 030
JAPKWR010000000			2,981,170	46.03	0.37	87.36	4.08	132	67,810	*Lachnospiraceae* bacterium, bin 031
JAPKWS010000000			1,729,729	42.39	0.17	71.37	13.5	254	30,173	*Clostridia* bacterium, bin 032
JAPKWT010000000	GCF_002933775.1	99.07	3,419,211	46.74	2.56	78.21	8.68	72	38,003	*Prevotella* sp., bin 033
JAPKWU010000000			2,679,807	44.95	0.57	95.34	2.42	339	14,766	*Odoribacter* sp., bin 034
JAPKWV010000000	GCA_002428825.1	98.53	1,914,688	59.72	0.95	98.32	1.04	224	87,352	*Alistipes* sp., bin 035
JAPKWW010000000	GCA_002494015.1	99.94	2,917,273	52.01	4.10	95.66	0.40	52	82,727	*Duncaniella* sp., bin 036
JAPKWX010000000	GCF_004803915.1	99.41	3,178,942	49.17	1.44	90.75	6.60	214	40,682	Duncaniella dubosii, bin 037
JAPKWY010000000			2,142,509	36.95	0.39	93.16	5.84	245	6,302	“*Candidatus* Gastranaerophilales” bacterium, bin 038
JAPKWZ010000000	GCF_003833075.1	96.53	3,771,089	49.04	10.7	99.43	2.70	60	53,253	*Muribaculum* sp., bin 039
JAPKXA010000000	GCA_003513705.1	99.50	2,077,509	46.87	0.51	66.96	1.95	481	5,638	*Muribaculaceae* bacterium, bin 040
JAPKXB010000000		98.85	5,554,479	38.32	5.66	98.05	3.74	751	15,829	*Lachnospiraceae* bacterium, bin 041
JAPKXC000000000	GCA_003979155.1		1,845,454	48.04	0.36	82.98	0.13	120	43,933	*Muribaculaceae* bacterium, bin 042
JAPKXD010000000	GCF_011959405.1	99.74	2,587,822	51.28	4.12	98.29	6.24	256	33,926	*Muribaculaceae* bacterium, bin 043
JAPKXE010000000	GCF_011959105.1	99.28	1,975,854	53.21	2.14	87.92	0.75	163	71,475	*Muribaculaceae* bacterium, bin 044
JAPKXF010000000		99.40	3,970,688	51.83	5.04	94.25	1.26	390	20,216	*Acetatifactor* sp., bin 045
JAPKXG010000000			1,552,516	26.73	0.37	93.26	2.81	492	87,467	*Bacillus* bacterium, bin 046
JAPKXH010000000			1,133,810	48.93	0.09	42.36	2.67	47	58,474	*Firmicutes* bacterium, bin 047
JAPKXI010000000	GCA_002362235.1		4,221,123	38.63	2.42	98.10	1.62	572	2,159	*Lachnospiraceae* bacterium, bin 048
JAPKXJ010000000		99.38	2,145,455	57.14	4.56	98.40	2.56	251	29,355	*Alistipes* sp., bin 049
JAPKXK010000000			1,606,242	28.19	0.47	91.01	1.12	630	19,231	*Bacillus* bacterium, bin 050
JAPKXL010000000			2,828,579	46.24	1.01	96.64	<0.01	20	185,047	*Oscillospiraceae* bacterium, bin 051

a All accession numbers are associated with BioProject accession number PRJNA876044.

b The closest genome as determined by average nucleotide identity (ANI) analysis. This and the closest reference genome are used to determine the species assignment.

c GTDB metric for fast alignment-free computation of the whole-genome ANI. This is calculated only when the query genome can be placed within a defined genus.

d Genome completeness calculated by CheckM.

e Contamination determined by CheckM.

f Total number of contigs in the MAG.

g Length of the shortest contig in the set of largest contigs making up at least 50% of the total assembly.

h NCBI-assigned taxonomy and original bin numbers from the KBase analysis.

### Data availability.

Raw metagenomic sequences and the MAGs can be found at NCBI under BioProject accession number PRJNA876044. Details on the analysis of the assemblies can be found as a KBase narrative (https://doi.org/10.25982/116829.47/1887425).
